# Advancements in metabolomics research in benign gallbladder diseases: A review

**DOI:** 10.1097/MD.0000000000038126

**Published:** 2024-05-24

**Authors:** Yanzhang Du, Wennie A. Wijaya, Wei Hui Liu

**Affiliations:** a Department of Gastroenterology, Sichuan Academy of Medical Sciences and Sichuan Provincial People’s Hospital, University of Electronic Science and Technology of China, Chengdu, China; b West China Hospital School of Medicine, Sichuan University, Chengdu, China.

**Keywords:** benign gallbladder disease, cholangitis, cholelithiasis, gallbladder, gallstone, metabolomic

## Abstract

The burgeoning field of metabolomics has piqued the interest of researchers in the context of benign gallbladder diseases, which include conditions such as gallbladder polyps, gallstones, and cholecystitis, which are common digestive system disorders. As metabolomics continues to advance, researchers have increasingly focused their attention on its applicability in the study of benign gallbladder diseases to provide new perspectives for diagnostic, therapeutic, and prognostic evaluation. This comprehensive review primarily describes the techniques of liquid chromatography-mass spectrometry, gas chromatography-mass spectrometry, and nuclear magnetic resonance and their respective applications in the study of benign gallbladder disease. Metabolomics has made remarkable progress in various aspects of these diseases, ranging from early diagnosis, etiological research, assessment of disease progression and prognosis, and optimization of therapeutic strategies. However, challenges remain in the field of metabolomics in the study of benign gallbladder diseases. These include issues related to data processing and analysis, biomarker discovery and validation, interdisciplinary research integration, and the advancement of personalized medicine. This article attempts to summarize research findings to date, highlight future research directions, and provide a reference point for metabolomics research in benign gallbladder disease.

## 1. Introduction

Metabolomics is a discipline that comprehensively analyzes the small-molecule metabolites of organisms or specific biological samples and reveals the functional status and complexity of biological systems by studying metabolite changes.^[1]^ Metabolomics emerged relatively late after proteomics, transcriptomics, and genomics and has a wide range of applications.^[2–5]^ Metabolites are relatively conserved in different biological systems, resulting in minimal differences between species. This universality of metabolomics techniques makes them applicable in various fields. Although metabolomics reflects the end result of biological processes, the information it contains is highly complex and requires rigorous data analysis. The prototype of metabolomics, called metabolic profiling, was originally proposed in the late 1940s by Roger Williams and colleagues, whereas the concept of metabolomics was introduced in the early 1970s by MG Horning and others.^[6,7]^

Metabolomics research encompasses several levels of analysis, including metabolite target analysis (metabonomics analysis), metabolic profiling analysis, and metabolic fingerprinting analysis. These approaches can be applied to the analysis of a variety of biological samples such as blood, urine, saliva, and tissue samples and cells.^[8]^ Metabolomics establishes a link between metabolites and biological gene function. Quantitative and qualitative analysis of metabolites in biological systems reveals the patterns of metabolic changes in organisms. This provides a solid basis for studying disease mechanisms, early diagnosis, evaluating treatment efficacy, and predicting prognosis.^[9]^

Benign gallbladder diseases mainly include cholelithiasis, gallbladder polyps, adenomyomatosis, and cholecystitis.^[10]^ Epidemiologic studies have identified risk factors for gallbladder disease, including age, gender, obesity, and genetic factors.^[4]^ Cholelithiasis is the most common gallbladder disease worldwide, with an increasing incidence over the years, leading to complications such as biliary colic, cholecystitis, and biliary obstruction.^[11]^ Gallbladder polyps are localized outpouchings of the gallbladder mucosa and can potentially transform into gallbladder carcinoma, requiring close surveillance.^[12]^ Cholecystitis is an inflammatory disease of the gallbladder and can be classified as acute or chronic. It can lead to severe complications such as abscesses, perforation, and peritonitis.^[13]^ The frequency and complexity of benign gallbladder disease make it a focus of clinical research.^[14]^

Imaging techniques play a critical role in differentiating benign from malignant gallbladder disease. Imaging features such as invasion of surrounding tissue, growth of large polypoid lesions, and limitations in diffusion-weighted imaging can help distinguish between benign and malignant lesions.^[15]^ In one study, various ultrasound findings were analyzed to determine their significance in differentiating gallbladder cancer from benign gallbladder disease. The study found that ultrasound features such as wall thickening, intraluminal mass, and pericholecystic fluid were more common in patients with gallbladder cancer than in patients with benign disease.^[16]^ Physical examination is also important for the diagnosis of benign gallbladder disease. A retrospective study evaluated the clinical findings, imaging results, preoperative ultrasound diagnosis, and histopathologic findings in patients with benign gallbladder disease. The study concluded that not all cases require histologic examination of the gallbladder. However, histologic examination may be helpful in cases with suspected malignancy or uncertain preoperative diagnosis.^[15]^

**Figure 1.**
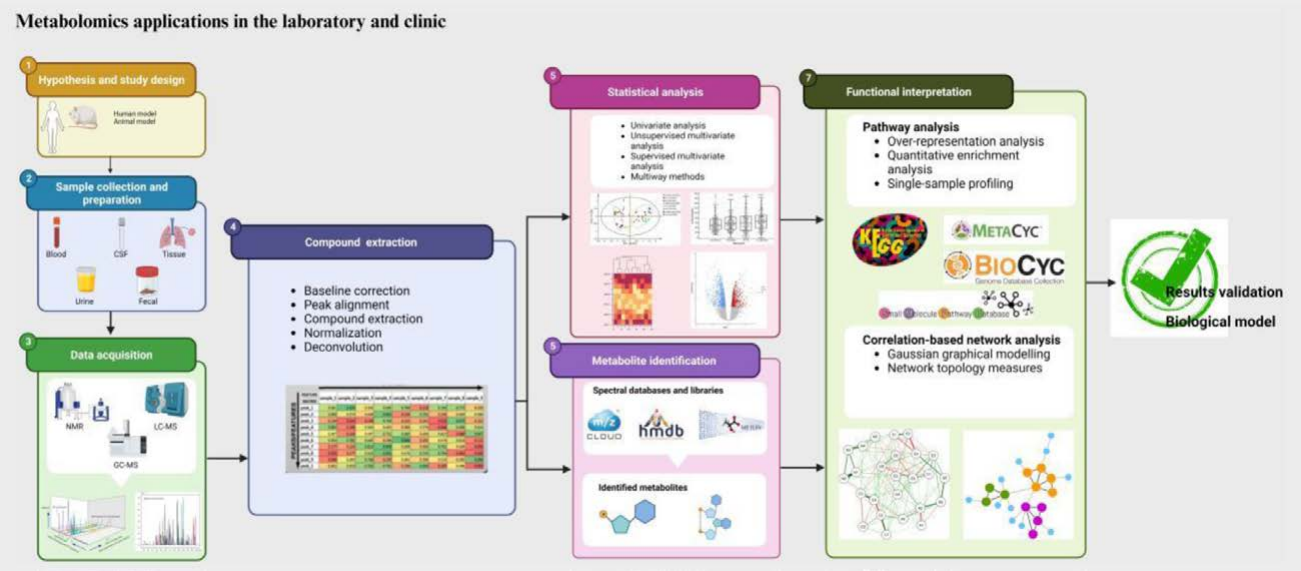
Analytical workflow of metabolomics studies.

The treatment approach for benign gallbladder disease depends on the circumstances. For example, in symptomatic patients with gallstones or acute cholecystitis, cholecystectomy is usually the standard treatment.^[4]^ In contrast, for small gallbladder polyps that are less than 1 centimeter in size and have no suspicious features on imaging studies, observation is often the best choice.^[17]^ Adenomyomatosis, when asymptomatic, is generally treated conservatively. In recent years, metabolomics, as an important branch of systems biology, has made remarkable progress in the study of benign gallbladder diseases, providing new insights into understanding disease mechanisms, diagnosis, and treatment. This review aims to summarize the progress and future prospects of metabolomics in the study of benign gallbladder diseases (Fig. 1).

## 2. Relevant sections

### 2.1. Development and applications of metabolomics techniques

The selection of a metabolomics analytical platform for investigating benign gallbladder diseases is dependent upon several aspects, such as the research objectives, sample characteristics, and the metabolites of concern. Below is an analysis of how various strategies are well-suited to particular conditions and applications:

#### 2.1.1. Liquid chromatography-mass spectrometry

Liquid chromatography-mass spectrometry (LC-MS) is a technique used to separate and analyze chemical compounds in a liquid sample by combining chromatography with mass spectrometry. Compared to nuclear magnetic resonance spectroscopy (NMR) and gas chromatography–mass spectrometry, LC-MS offers higher sensitivity, relatively simple sample preparation, and a wide range of analytes. It is an efficient, sensitive and reproducible method for the analysis of low molecular weight metabolites in plasma and urine. LC-MS is an essential analytical platform in metabolomics research, especially in untargeted metabolomics analysis. With the rapid development of ultra-performance liquid chromatography and Fourier transform ion cyclotron resonance mass spectrometry as detection methods, LC-MS has gained significant advantages. Consequently, LC-MS is widely used in metabolomics research compared with other techniques.^[18,19]^

*Suitable conditions*: LC-MS exhibits exceptional adaptability and is capable of examining a broad spectrum of metabolites, encompassing both polar and nonpolar substances. This renders it well-suited for investigating the varied metabolic changes linked to gallbladder diseases.

##### 2.1.1.1. Applications

1. *Untargeted profiling:* LC-MS is employed in non-targeted metabolomics to detect and measure a wide range of metabolites in bile samples, offering a thorough understanding of metabolic alterations linked to gallbladder illnesses.
2. *Targeted analysis:* LC-MS can be used to accurately measure and compare specific metabolites of interest, like bile acids or lipid species, in bile samples, enabling precise quantification and comparison of various disease states.

#### 2.1.2. Gas chromatography-mass spectrometry

Gas chromatography-mass spectrometry (GC-MS) is a technique used to separate and analyze the components of a mixture based on their molecular characteristics. GC-MS is considered one of the best options for the analysis of various compounds, including lipids, drug metabolites, and environmental pollutants. It offers high sensitivity, high resolution, and a wide analytical range, making it widely used in metabolomics research.^[20]^ GC-MS allows identification of the detected species based on retention time and mass spectrum and has the advantage of obtaining fragment spectra that are unaffected by instrumental factors.^[21]^ In metabolomics research, GC-MS is one of the most efficient, reproducible, and widely used analytical platforms suitable for untargeted, semitargeted, and targeted approaches.^[22]^ It can identify and quantify small molecule metabolites and discover new compounds. However, prior to GC-MS analysis, metabolite extracts must be derivatized to ensure volatility and thermal stability for detection. This requirement somewhat limits the development of the technique.^[23,24]^

*Suitable conditions*: GC-MS is highly ideal for the analysis of volatile and thermally stable metabolites, making it well-suited for the investigation of certain categories of metabolites present in bile samples, such as fatty acids and select bile acids.

##### 2.1.2.1. Applications

*Targeted analysis*: GC-MS is frequently employed to perform focused analysis of particular metabolites following derivatization, such as bile acids, fatty acids, and other volatile metabolites. This technique offers exceptional sensitivity and repeatability.

#### 2.1.3. Nuclear magnetic resonance

NMR is one of the earliest and most widely used techniques in metabolomics.^[25]^ It is based on the spin properties of atomic nuclei, where the nuclei absorb radiation in an external magnetic field, resulting in energy conversion. NMR has advantages, such as small sample volumes, no sample preparation, and nondestructive and noninvasive detection. In addition, NMR spectra can be obtained rapidly and noninvasively from specific regions of a biological organism. As a noninvasive metabolomics analysis method, it has good reproducibility and quantification, making it suitable for the study of benign gallbladder diseases.^[26]^ Studies have used NMR-based metabolomics approaches to investigate metabolic changes in the serum of patients with chronic cholecystitis, establishing the application of NMR in disease diagnosis and detection of metabolic patterns in biological fluids.^[27]^ However, NMR also struggles with low sensitivity, which makes it difficult to detect low abundance compounds, limiting its application to some extent.^[28]^

*Suitable conditions*: NMR spectroscopy is nondestructive and requires minimal sample preparation, making it suitable for analyzing intact bile samples or biofluids with minimal processing.

##### 2.1.3.1. Applications

1. *Untargeted profiling*: ^1^H-NMR spectroscopy is often used for non-targeted metabolomics analysis of bile samples, providing quantitative information on a wide range of metabolites without the need for chromatographic separation.
2. *Structural elucidation*: NMR spectroscopy can provide structural information about metabolites, aiding in the identification and characterization of novel biomarkers or metabolites associated with gallbladder diseases.

#### 2.1.4. High-performance liquid chromatography

High-performance liquid chromatography (HPLC) is a technique used to separate, identify, and quantify components in a mixture based on their interactions with a stationary phase and a mobile phase.

*Suitable conditions*: HPLC techniques are especially advantageous for the separation and quantification of distinct categories of metabolites, such as bile acids and bilirubin, in samples of bile.

##### 2.1.4.1. Application

*Targeted analysis*: HPLC techniques combined with different detectors such as UV-Vis and fluorescence can be employed to accurately measure particular metabolites that are important for gallbladder illnesses. This approach offers excellent sensitivity and selectivity.

### 2.2. Other related techniques

*Surface desorption atmospheric pressure chemical ionization mass spectrometry* allows direct analysis of samples that are in a gaseous environment. It uses surface desorption techniques to volatilize analytes from the sample, followed by chemical reactions to ionize the volatiles and analyze them by mass spectrometry. Surface desorption atmospheric pressure chemical ionization mass spectrometry has good selectivity and sensitivity, making it suitable for rapid detection of drug metabolites and analysis of chemical components.^[29]^

*Paper spray ionization mass spectrometry* is a simple, rapid, and inexpensive technique for direct mass spectrometry. It involves spraying the sample directly onto a piece of paper, followed by ionization of the analytes using an ionization source and subsequent mass spectrometric analysis. Paper spray ionization mass spectrometry is suitable for the analysis of small molecular compounds in biological samples, such as drug metabolites and amino acids.^[30]^

These methodologies aid in the improvement of metabolomic research with enhanced selectivity and sensitivity, as does the continuous development of instruments, data analysis tools, and sample preparation strategies. In order to handle the complexity of biological systems and provide new insights into metabolism and disease causes, researchers continue to investigate and refining these techniques. In the context of disease-focused metabolomics research, the use of green biomaterials offers several advantages, particularly in terms of biocompatibility, sustainability, and environmental impact. By using these eco-friendly nanomaterials in disease-focused metabolomics research, researchers can lessen the environmental impact of their work and contribute to the development of sustainable analytical methodologies.^[31]^

In short, the selection of a metabolomics analytical platform to investigate benign gallbladder diseases is contingent upon the particular research goals, sample characteristics, and target metabolites. The incorporation of various analytical platforms or techniques can yield supplementary data and augment the overall comprehension of the metabolic changes linked to gallbladder diseases.

### 2.3. The choice of sample for metabolomics analysis

As well as the availability of samples and the particular metabolites of interest, the selection of a sample for metabolomics analysis in benign gallbladder diseases is contingent on a number of additional factors. As per the following applications, suggestions are provided for sample selection:

#### 2.3.1. Serum

Serum samples can be used in systemic metabolomics studies to better understand the metabolic changes associated with benign gallbladder disease. They can shed light on systemic metabolic changes and potentially serve as biomarkers for disease diagnosis or prognosis.

*Advantages*: Serum samples are relatively simple to collect using minimally invasive techniques like venipuncture. They provide a snapshot of systemic metabolism and can be applied to large-scale epidemiological studies or clinical trials.

Factors other than gallbladder disease that may influence serum metabolites include diet, medication, and other systemic conditions. When analyzing and interpreting data, potential confounders should be carefully considered.

#### 2.3.2. Bile samples

Bile samples are directly related to gallbladder physiology and pathology, making them ideal for studying local metabolic changes and bile composition changes caused by benign gallbladder diseases.

*Advantages*: Bile samples are a direct reflection of bile metabolism and can reveal disease-specific changes in bile composition, such as changes in bile acids, lipids, and other metabolites.

*Considerations*: Obtaining bile samples may necessitate invasive procedures such as endoscopic retrograde cholangiopancreatography or percutaneous transhepatic cholangiography. Furthermore, bile samples may be limited in volume and vary in composition due to factors such as fasting status and gallbladder function.

#### 2.3.3. Fecal extract

Fecal samples are useful for investigating gut microbial metabolism and the gut-liver axis in benign gallbladder diseases. They can shed light on changes in gut microbiota composition and function associated with gallbladder diseases.

*Advantages*: Fecal samples are noninvasive and can be collected longitudinally to track changes in gut microbial metabolism. They provide a comprehensive overview of microbial-host metabolic interactions.

*Considerations*: Fecal metabolites may reflect both host and microbial metabolism, necessitating careful analysis of results. Standardized collection and storage procedures are required to reduce variation in fecal metabolite profiles.

#### 2.3.4. Gallstone

Gallstones can be analyzed to learn about their composition as well as the metabolic processes that lead to their formation and dissolution. Metabolomics analysis of gallstones can reveal lipid and mineral components involved in gallstone pathogenesis.

*Advantages*: Gallstones provide direct insight into the composition of solid materials within the gallbladder and can reveal metabolic pathways involved in gallstone formation.

*Considerations*: Obtaining gallstone samples may necessitate surgical procedures such as cholecystectomy. Furthermore, gallstone composition can differ between individuals and disease states, necessitating careful characterization and standardization of sample handling procedures.

#### 2.3.5. Bile sample biopsy

Biopsy samples collected during endoscopic procedures can provide detailed information about gallbladder tissue metabolism and pathology. Metabolomics analysis of bile sample biopsies can reveal tissue-specific metabolic changes associated with benign gallbladder diseases.

*Advantages*: Bile sample biopsies allow for the investigation of tissue-specific metabolic changes and can reveal disease mechanisms at the molecular level.

*Considerations*: Collecting bile sample biopsies may necessitate specialized endoscopic techniques and expertise. When collecting biopsy samples, care should be taken to consider ethical and safety concerns.

In summary, the choice of sample for metabolomics analysis in benign gallbladder diseases depends on the specific research objectives and the type of metabolic alterations being investigated. Integration of multiple sample types and analytical platforms can provide complementary information and enhance the understanding of metabolic changes associated with gallbladder diseases.

### 2.4. Spectra of the typical benign biliary diseases

Benign biliary diseases include a variety of diseases affecting the bile ducts and gallbladder. While individual peak assignments in spectra can vary depending on the nature of the disease and the analytical technique utilized (e.g., nuclear magnetic resonance spectroscopy, mass spectrometry), the following are some common peak assignments that may be seen in spectra of benign biliary diseases:

1. *Bile acids*: Peaks for bile acids such cholic acid, chenodeoxycholic acid, deoxycholic acid, and lithocholic acid may be seen. In proton NMR spectra, these peaks are commonly seen between 0.5 and 3 ppm, whereas in mass spectra, they can be found between 300 and 500 m/z.^[32–36]^
2. *Bilirubin*: Peaks linked with bilirubin, a byproduct of heme metabolism, may exist. Bilirubin peaks can be found about 2.5 to 2.8 ppm in proton NMR spectra and around m/z 500 to 600 in mass spectra.
3. *Phospholipids*: Peaks for phospholipids, such as phosphatidylcholin and phosphatidylethanolamine, may be visible. In proton NMR spectra, these peaks normally appear between 3.2 and 4.3 ppm, whereas in mass spectra, they appear between 600 and 900 m/z.^[37–39]^
4. *Cholesterol*: Spectra may exhibit peaks that correlate to cholesterol. Cholesterol peaks often manifest at approximately 0.6 to 0.9 parts per million (ppm) in proton nuclear magnetic resonance (NMR) spectra and at mass-to-charge ratio (m/z) values of 400 to 450 in mass spectra.^[27,40]^
5. *Biliary salts*: Peaks related to biliary salts, such as glycocholic acid and taurocholic acid, may be seen. These peaks commonly manifest within the range of 0.7 to 1.2 ppm in proton NMR spectra or within the m/z range of 400 to 600 in mass spectra.^[38,41,42]^
6. *Amino acids and metabolites*: Peaks representing amino acids, organic acids, and other metabolites may be seen, indicating the metabolic changes linked to biliary disorders. The chemical shifts or mass-to-charge ratios of these peaks can exhibit significant variations.^[39,43,44]^

It is crucial to acknowledge that the specific arrangement of peaks and their designations may differ based on factors such as the particular disease condition, techniques utilized for sample preparation, and the sensitivity and resolution of the analytical technique employed. For precise diagnosis and characterization of benign biliary disorders, it is essential to analyze spectra alongside clinical and biochemical data.

### 2.5. Methods for data processing and statistical analysis

Metabolomics data processing and statistical analysis are critical steps in metabolomics research. Typically, both unsupervised and supervised learning methods are used. In unsupervised learning methods, principal component analysis is a commonly used approach that reveals the linear combinations of variables that contribute to the variance in the data matrix.^[45]^ In supervised learning methods, partial least squares discriminant analysis and orthogonal partial least squares discriminant analysis (OPLS-DA) are commonly used. These methods establish models to relate metabolite expression levels to sample categories and allow prediction of sample categories.^[46]^ Among them, OPLS-DA has better interpretability and predictive ability, allowing a better understanding of variables related to biological processes. Therefore, the selection of appropriate methods for metabolomics data processing and statistical analysis is crucial because they can effectively reveal biological significance in metabolomics research.^[47,48]^

### 2.6. Metabolomics in the study of benign gallbladder disease

#### 2.6.1. Metabolomics in gallbladder polyps research

he application of metabolomics in the study of gallbladder polyps is primarily focused on early diagnosis and etiologic research. By analyzing metabolites in biological samples such as plasma and urine from patients with gallbladder polyps, researchers have successfully discovered various biomarkers associated with gallbladder polyps. In a study comparing bile acids in patients with gallbladder polyps, healthy individuals, patients with hepatocellular carcinoma, and patients with gallbladder cancer, lipidomic analysis of 9 lipid categories (carnitines, ceramides, diacylglycerols, fatty acids, lysophosphatidylcholines, lysophosphatidylethanolamines, phosphatidylcholines, phosphatidylethanolamines, and triglycerides) was performed. Phosphatidylcholines (PCs) were the most conspicuous. Differences in the intensity of these 6 phosphatidylcholines were found between the gallbladder polyp, healthy, and hepatocellular carcinoma groups, suggesting that lipid metabolism may not be closely associated with the occurrence and development of gallbladder polyps (Table 1).^[37]^

**Table 1:** Metabolomics research on benign gallbladder diseases.

Author	Analytical platform	Robust data analysis methods	Quality of the model/fitting	Subjects	Samples	Differential metabolites/metabolic pathways	Statistic	Objective of the study
*Gallbladder polyp*								
Jang et al 2023^[37]^	LC-MS/MS	n/a	n/a	Healthy subjects (n = 37) Gallbladder polyp (n = 18) Hepatocellular carcinoma (n = 6) Gallbladder cancer (n = 8)	Bile	Carnitines, ceramides, diacylglycerols, fatty acids, lysophosphatidylcholines, lysophosphatidylethanolamines, phosphatidylcholines, phosphatidylethanolamines, and triglycerides	*t* test, *P* < .05	The study of 37 bile samples’ IR spectra revealed distinct characteristics for each separated phase, allowing GB cancer samples to be distinguished from normal, GB polyp, and HCC samples. However, because the bile compositions of HCC and normal/GB polyp samples were identical, differentiation was not possible. To establish clinical value, future studies should investigate cholesterol and proteins with a bigger sample size
Zhao et al 2016^[32]^	HPLC-TOF/MS	n/a	n/a	Cholesterol polyp (n = 18) Adenomatous polyp (n = 9) Gallstone (n = 20)	Bile and blood	Glycocholic acid, taurocholic acid, glycochenodeoxycholic acid, taurochenodeoxycholic acid, glycodeoxycholic acid, taurodeoxycholic acid, taurolithocholic acid, tauroursodeoxycholic acid	*t* test, *P* < .05	Serum GCA, GCDCA, and TCDCA levels were higher in patients with adenomatous polyps than cholesterol polyps, suggesting their accumulation could differentiate between the 2. HPLC technology could simplify and convenient diagnosis of gallbladder polypoid lesions
*Gallstone*								
Albiin et al 2008^[33]^	^1^-H NMR spectroscopy	n/a	n/a	Common bile duct stone (n = 6)	Bile	Phosphatidylcholine, taurine, lipid/lactate, cholesterol/lipid, cholesterol	Conventional analysis (peak intensity/area analysis), Computerized multivariate analysis (statistical classification strategy) *t* test, *P* < .05	The ^1^H-MRS of bile can assist distinguish cholangiocarcinoma from benign biliary diseases, with or without PSC
Srivastava et al 2008^[40]^	^1^-H NMR spectroscopy	n/a	n/a	GBC (n = 11), Chronic cholecystitis (n = 23), Xanthogranulomatous cholecystitis (n = 11)	Gallstone	Cholesterol, calcium, and magnesium	*t* test, *P* < .05	The composition of gallbladder stones (GS) can reveal the etiopathogenesis of gallbladder cancer, aiding in the identification of patients at high risk of developing GBC and advocating for prophylactic cholecystectomy
Ijare et al 2010^[34]^	^1^-H NMR spectroscopy	n/a	n/a	Suspected CBD gallstones (n = 1), CBD gallstones + duodenal ulcers (n = 1), Pancreatitis (n = 3), Pancreatic cancer (n = 4)	Bile	Glycerin-conjugated bile acids (GCBAs), taurine-conjugated bile acids (TCBAs), total bile acids (TBAs), and choline-containing phospholipids (choline-PLs)	n/a	The method enables simultaneous quantification of glycine- and taurine conjugated bile acids, determining their conjugation pattern, which could aid in noninvasive diagnostics in in vivo settings, using clinical scanners for 1H MRS of gallbladder bile
Chen et al 2018^[35]^	HPLC-TOF/MS	OPLS-DA	*R*^2^ = 0.168	Gallstone (n = 30), Healthy subjects (n = 30)	Blood	Ursodeoxycholic acid (UDCA) and up-regulated differential trend metabolites GCA, HDCA, TCA, GDCA, glycine chenodeoxycholic acid (GCDCA), HCA, taurine chenodeoxycholic acid (TCDCA), and 7-ketodeoxycholic acid (7-ketoDCA)	*t* test, *P* < .05	HDCA has the potential to predict the progression of gallstone disease by analyzing the incidence of gallstones
Ma et al 2018^[43]^	^1^-H NMR spectroscopy	OPLS-DA, PCA	Blood: *R*^2^ Y = 90.3%, Q^2^ = 58.9%; Bile: *R*^2^ Y = 84.1%, Q^2^ = 74.8%	Gallbladder stone: serum samples (n = 17), bile samples (n = 19); Liver transplantation donor: serum samples (n = 10), bile samples (n = 15)	Blood, bile	Valine, alanine, lysine, glutamine, glutamate, pyruvate, creatinine, choline, alpha-glucose, beta-glucose, tyrosine, histidine, and hypoxanthine, 1, 2-propanediol, acetoacetate, and lactate	*t* test, *P* < .05	^1^H-NMR metabonomics reveals significant differences in serum and bile metabolites between gallbladder stone patients and healthy men, aiding in the investigation of gallbladder stone pathogenesis
Wu et al 2020^[36]^	LC-MS/MS	PCA	n/a	13 healthy controls, 292 cholecystolithiasis and 25 non-neoplastic polyps	Blood	Ursodeoxycholic acid (UDCA), TUDCA, GUDCA, taurochenodeoxycholic acid (TCDCA), and glycine-chenodeoxycholic acid (GCDCA)	*t* test, *P* < .05	The study found significant changes in bile acid composition in cholecystolithiasis and nonneoplastic polyps, particularly in UDCA, TUDCA, GUDCA, TCDCA, and GCDCA. These changes may contribute to the development of gallbladder diseases, as bile acids can affect intestinal microbial community structure, estrogen influence on cholesterol metabolism, and risk factors like hypertension and obesity. Future research should address imbalances in subjects
Zdanowicz et al 2022^[38]^	UHPLC/MS/MS	n/a	n/a	Pediatric patients: Gallstone (n = 48), Controls (n = 38)	Blood	Sphingolipids, including C16:0-LacCer, C18:0-LacCer, C18:1-LacCer, C24:0-LacCer, C24:1-LacCer, C14:0-Cer, C14:0- Cer, C16:0-Cer, C18:0-Cer, C18:1-Cer, C20:0-Cer, C22:0-Cer, C24:0-Cer, C24:1-Cer, SPA and Sph	ROC (AUC = 1.0)	Serum sphingolipids may serve as biomarkers for cholelithiasis patients, but further research is needed to determine if these lipids regulate gallstone formation and if abnormal ceramide levels predispose patients
*Cholecystitis*								
Sharma et al 2017^[27]^	^1^-H NMR spectroscopy	PCA, PLS-DA	*R*^2^ = 0.89, Q^2^ = 0.78	Chronic cholecystitis (n = 41) Control (n = 30)	Blood	Glutamine, LDL, VLDL, BCAA, histidine, and tyrosine, whereas the levels of formate, lactate, and 1,2-propanediol	ROC (AUC = 0.99)	NMR aids in disease diagnosis and metabolic pattern recognition in biofluids, aiding in early diagnosis of gallstone inflammation, a major cause of gallbladder cancer
Li et al 2012^[39]^	^1^-H NMR spectroscopy	PCA, PLS-DA	n/a	Rabbit model: Acalculous cholecystitis (n = 7) Control (n = 7)	Blood	Phospholipids, lactic acid, 3-hydroxybutyric acid, lysine, citric acid, asparagine, histidine, glucose, etc	OPLS-DA	The study found significant differences in serum endogenous small-molecule metabolites between rabbits with acute acalculous cholecystitis and healthy controls, but limitations include needing further validation and clinical research support
*Cholangitis*								
Hao et al 2021^[41]^	LC-MS/MS	OPLS-DA	*R*^2^Y = 0.876 and Q^2^ = 0.479	Choledocholithiasis with concomitant cholangitis (n = 19), Healthy control (n = 19)	Feces	KYNA, tryptophan metabolism, N-palmitoylsphingosine, neuroactive sphingolipid, ceramide signaling pathways	*t* test, *P* < .05	This study offers new perspectives on changes in the composition and function of the gut microbiome and metabolite profiles in CC, as well as the pathways linking gut microbiota and biliary inflammation
Hao et al 2022^[42]^	LC-MS/MS	OPLS-DA	*R*^2^Y = 0.876; Q^2^ = 0.479	Choledocholithiasis with concomitant cholangitis (n = 25), Healthy control (n = 25)	Fresh tail stool	Biofilm formation, lipopolysaccharide synthesis, propionate metabolism, and glutathione metabolism	*t* test, *P* < .05	The study reveals gut microbiome changes in patients with Choledocholithiasis with concomitant cholangitis, potentially contributing to the development of the condition, aiming for prevention and therapeutic interventions
Zhang et al 2023^[44]^	UHPLC-MS	n/a	n/a	280 patients with acute cholangitis: mild group (n = 65), moderate group (n = 84), and severe group (n = 131) based on the TG18 criteria	Blood	Procalcitonin and plasma acylcarnitines	ROC (AUC = 0.893)	Presepsin could predict acute cholangitis severity and biliary drainage need, while acetyl-L-carnitine could be a prognostic factor. Innate immune response linked to mitochondrial metabolic dysfunction

BCAA = alanine, branched-chain amino acids, CC = chronic cholecystitis, GCA = glycinecholic acid, GDCA = glycine deoxycholic acid, GUDCA = glycoursodeoxycholic acid, HCA = hyodeoxycholic acid, HDCA = deoxycholic acid, HPLC = high-performance liquid chromatography, KYNA = kynurenic acid, LC-MS = liquid chromatography-mass spectrometry, LDL = low-density lipoprotein, NMR = nuclear magnetic resonance spectroscopy, OPLS-DA = orthogonal partial least squares discriminant analysis, PCA = Principal Component Analysis, PLS-DA = partial least square discriminant analysis, SPA = sphingomyelin, TCA = taurocholic acid, TCDCA = taurine chenodeoxycholic acid, TUDCA = tauroursodeoxycholic acid, VLDL = very low-density lipoprotein.

Differentiating between cholesterol polyps (CPs) and adenomatous polyps (APs) has been a clinical challenge. Using HPLC as the analysis platform, the concentrations of glycine chenodeoxycholic acid (GCDCA) and taurine chenodeoxycholic acid (TCDCA) in gallbladder bile were significantly higher in the CP group than in the AP group, while the concentrations of GCDCA, TCDCA and glycinecholic acid (GCA) in serum were significantly higher in the AP group than in the CP group. GCDCA in serum alone showed relatively good diagnostic performance. Therefore, the concentrations of GCA, GCDCA, and TCDCA in serum may be valuable for the differential diagnosis of APs and CPs (Table 1).^[32]^

#### 2.6.2. Metabolomics in gallstone research

The application of metabolomics methods in gallstone research is primarily focused on early diagnosis and the study of etiology. By analyzing metabolites in biological samples such as plasma, urine, and bile from patients with gallstones, researchers have discovered a number of biomarkers associated with stone formation. These biomarkers primarily involve metabolic pathways related to cholesterol, bile acids, lipid metabolism, and mineral metabolism,^[49]^ providing insights into the pathogenesis and preventive measures of gallstones (Table 1).

Taurine and glycine are 2 amino acids that can conjugate with bile acids to form bile salts, which are essential for emulsifying and absorbing dietary fats and fat-soluble vitamins. The balance of taurine and glycine conjugated bile acids is highly regulated, and disruptions in this balance can indicate underlying hepatobiliary dysfunction. While changes in the ratio of taurine to glycine conjugated bile acids have been linked to a variety of biliary diseases, including cancer, there is little direct evidence that this particular change is unique to benign biliary diseases. Bile acid metabolism and composition are complex and can be influenced by a variety of factors, such as genetic predisposition, dietary habits, and underlying pathophysiologic mechanisms.

Several studies have looked into changes in the taurine to glycine conjugated bile acid ratio in the context of different hepatobiliary diseases. Nagana Gowda et al^[50]^ conducted a study that contributes significantly to our understanding of bile acid metabolism and composition, particularly in the context of biliary diseases.

This study describes a single-step method for analyzing individual conjugated bile acids in human bile using ^1^H NMR spectroscopy. The researchers used this technique to quantify specific bile acid species, including taurine and glycine conjugates, in human bile samples. This methodology offers a comprehensive approach to bile acid composition research, allowing for simultaneous measurement of multiple bile acid species.

While the reference does not directly address changes in the ratio of taurine to glycine conjugated bile acids in biliary diseases, it does lay the groundwork for future research by providing a reliable analytical method for bile acid analysis. Later in the year, Ijare et al^[34]^ present a study that uses 1H NMR spectroscopy to simultaneously quantify glycine- and taurine-conjugated bile acids, total bile acids, and choline-containing phospholipids in human bile (Table 1). This methodology provides a comprehensive approach to analyzing bile components, including bile acids, which are critical for understanding the pathophysiology of biliary diseases. While the study does not directly address changes in the ratio of taurine to glycine conjugated bile acids in biliary diseases, it does provide a useful methodological framework for studying bile acid composition, which is important for understanding the metabolic changes associated with these diseases. Further research with a large patient cohort is needed to establish a definitive correlation between bile acid conjugate levels and hepatobiliary disease type.

Albiin et al^[33]^ discuss the potential of magnetic resonance spectroscopy (MRS) in diagnosing cholangiocarcinoma, a type of bile duct cancer, in patients with and without primary sclerosing cholangitis (PSC). This study focuses on the use of MRS in analyzing the metabolic profile of bile to detect cholangiocarcinoma, a difficult diagnosis due to nonspecific symptoms and the limited sensitivity of conventional imaging techniques. The researchers used MRS to examine the metabolic composition of bile in order to identify characteristic metabolic alterations associated with cholangiocarcinoma, which could aid in diagnosis (Table 1).

Srivastava et al^[40]^ conducted a study using samples of stones dissolved in organic solvents from patients with gallbladder cancer (GBC) and benign diseases, including chronic cholecystitis (CC) and xanthogranulomatous cholecystitis. They measured cholesterol, calcium, and magnesium with ^1^H-NMR. The results showed significantly lower cholesterol in GBC compared with the benign disease group. Calcium and magnesium were significantly higher in GBC compared to the benign disease group. Calcium content was higher than magnesium content in all gallstones. Calcium and magnesium showed a positive correlation in GBC and xanthogranulomatous cholecystitis, while cholesterol and calcium showed a negative correlation in CC. The implications of these results hold significant clinical value as they indicate that the composition of gallstones may serve as a potential biomarker for differentiating benign conditions from gallbladder cancer (Table 1). Through the integration of molecular markers evaluated via laboratory tests and imaging findings acquired from abdominal ultrasound, CT, or MRI, medical professionals can augment their capacity to distinguish between benign biliary diseases and gallbladder cancer. This multidisciplinary approach may enhance the precision of patient diagnoses and direct the development of effective treatment plans.

In another study, metabolomics techniques were used to determine the metabolic profile of bile acids in the serum of gallstone patients. They found a down-regulated differential metabolite ursodeoxycholic acid (UDCA) and up-regulated differential trend metabolites GCA, deoxycholic acid (HDCA), Taurocholic acid, glycine deoxycholic acid, GCDCA, hyodeoxycholic acid, TCDCA, and 7-ketodeoxycholic acid. HDCA has been identified as a bile acid that may predict gallstone formation throughout the disease process (Table 1).^[35]^ A study conducted at the West China Hospital, Sichuan University found that patients with gallstones had significantly decreased concentrations of valine, proline, lysine, glutamine, glutamic acid, pyruvic acid, creatinine, choline, α-glucose, β-glucose, tyrosine, histidine, and xanthine compared with healthy individuals without gallstones, whereas the concentrations of lactate, acetic acid, and 1,2-propanediol were significantly increased. The study elucidated the mechanisms underlying the pathogenesis of gallstones based on the amino acid and glucose metabolism profiles (Table 1).^[43]^

Wu et al^[36]^ conducted a study using LC-MS to compare plasma bile acid (BA) profiles between patients with GBD, such as gallstones and non-neoplastic polyps, and a healthy control group. The results showed that the burden of GBD was higher in women than in men (63.36% for gallstones, 60% for non-neoplastic polyps). Gallstones and non-neoplastic polyps were associated with increased secondary BAs in plasma, while primary BA levels were lower than in the healthy control group. BAs such as UDCA, tauroursodeoxycholic acid, glycoursodeoxycholic acid, taurochenodeoxycholic acid (TCDCA), and glycine-chenodeoxycholic acid (GCDCA) were significantly decreased in the plasma of patients with gallbladder disease. UDCA showed a negative correlation with white blood cell count and the percentage of neutrophils. This study suggests that patients with gallstones and non-neoplastic polyps have higher levels of secondary BAs and that white blood cell count and percentage of neutrophils are negatively correlated with UDCA, suggesting that UDCA has some degree of anti-inflammatory effects.^[36]^

Lipid metabolism is an important component of metabolomics. In a prospective study, serum lipid metabolism was investigated in pediatric patients with cholelithiasis. The results showed significant differences in concentrations of total cholesterol (TC), sphingomyelin, sphingolipids (C14:0-Cer, C16:0-Cer, C18:1-Cer, C18:0-Cer, C20:0-Cer, and C24:1-Cer) and lactosylceramides (C16:0-LacCer, C18:0-LacCer, C18:1-LacCer, C24:0-LacCer, and C24:1-LacCer) between patients with cholelithiasis and those without cholelithiasis. After adjusting for age, sex, obesity, TC, and triglyceride (TG) levels, the study found that patients with cholelithiasis had lower concentrations of sphingomyelin, C14:0-Cer, C16:0-Cer, C24:1-LacCer, and C24:0-LacCer in their serum, whereas the concentrations of C20:0-Cer, C24:1-Cer, C16:0-LacCer, and C18:1-LacCer were higher. When the area under the curve was analyzed, C16:0-Cer was found to have the highest specificity and sensitivity. This suggests that C16:0-Cer can be used as a potential biomarker to determine whether cholelithiasis is present in children, thus supporting the auxiliary diagnosis in pediatric patients (Table 1).^[38]^ The potential utilization of sphingolipids C16:0-Cer as a biomarker for cholelithiasis in adult individuals appears beneficial, considering the parallels between the pathophysiology of gallstone formation in children and adults. However, additional research is required to confirm the effectiveness of sphingolipids C16:0-Cer as a biomarker in adult populations. To diagnose cholelithiasis in adults, it is necessary to conduct studies in adult groups to evaluate the sensitivity, specificity, and predictive value of sphingolipids C16:0-Cer. Moreover, evaluating the expenses linked to integrating sphingolipids C16:0-Cer into regular examinations necessitates taking into account multiple factors, such as laboratory costs, infrastructure needs, and healthcare system considerations.

#### 2.6.3. Metabolomics in cholecystitis research

Cholecystitis is another common benign disease of the gallbladder. By analyzing metabolites in biological samples such as plasma, urine, and bile from patients with acute cholecystitis, researchers have identified a number of biomarkers associated with an inflammatory response. Multivariate statistical analysis methods were used to detect and identify the major metabolites involved in cholecystitis. Patients with chronic cholecystitis were found to have decreased levels of serum metabolites such as glutamine, low-density lipoprotein (LDL), very low-density lipoprotein, alanine, branched-chain amino acids, histidine, and tyrosine, whereas the levels of formate, lactate, and 1,2-propanediol were increased (Table 1).^[27]^ These biomarkers are related to metabolic pathways involving lipid metabolism, amino acid metabolism, and inflammatory mediators,^[51]^ providing insights into the pathogenesis of cholecystitis and enabling personalized treatment. In experimental studies with modeled rabbits, nuclear magnetic resonance spectroscopy combined with multivariate statistical analysis was used to investigate serum metabolites in acute acalculous cholecystitis. It was observed that during cholecystitis, serum lipid levels are increased, whereas levels of phospholipids as well as low molecular weight metabolites such as lactate, 3-hydroxybutyrate, arginine, citrate, aspartate, histidine, and glucose are reduced. Analysis of these biological markers revealed that the major metabolic changes in the body induced by acute acalculous cholecystitis occur in the bile. The disruption of these metabolites involves lipids, proteins, and carbohydrates, indicating that the body’s metabolism is severely impaired during the inflammatory response (Table 1).^[39]^

#### 2.6.4. Metabolomics in cholangitis research

Cholangitis is a severe and potentially life-threatening infection of the intrahepatic and extrahepatic bile ducts, characterized by significant inflammation and swelling, often requiring early intervention. The most common causes of cholangitis are choledocholithiasis (common bile duct stones) and benign or malignant strictures.^[52]^ In one study, stool samples from patients with choledocholithiasis with concomitant cholangitis (CC) were subjected to LC-MS metabolomic analysis. The study revealed changes in the fecal metabolomic profile of CC patients. Reduced levels of kynurenic acid, involved in tryptophan metabolism, and increased levels of N-palmitoylsphingosine, involved in neuroactive sphingolipid and ceramide signaling pathways, were closely associated with progression of CC (Table 1).^[41]^

The gut microbiota and its metabolites may play an important role in the pathogenesis of choledocholithiasis associated with cholangitis. In one study, the changes in gut microbiota and metabolites were investigated in CC patients compared with healthy controls. Here, the gut microbiota community was defined by metagenomic sequencing and the metabolite profile was characterized by liquid chromatography-mass spectrometry (LC-MS). In the study, a significant reduction in fecal microbial community diversity was observed in CC patients. In addition, significant alterations in several metabolic pathways were observed in CC patients, including those involved in biofilm formation, lipopolysaccharide synthesis, propionate metabolism, and glutathione metabolism. The study also identified 47 significantly altered metabolites, including substances related to tryptophan metabolism and sphingolipid signaling pathways. These findings reveal alterations in the composition and function of the gut microbiota in CC patients and their possible mechanisms in bile duct inflammation (Table 1).^[42]^

The prognosis of acute cholangitis is of paramount importance. Zhang et al^[44]^ used chemiluminescence enzyme immunoassay and ultra-high performance liquid chromatography-mass spectrometry to measure procalcitonin and plasma acylcarnitines in the blood of patients with acute cholangitis. They found that the concentrations of procalcitonin, short-chain acylcarnitines, and medium-chain acylcarnitines increased with disease severity, whereas the concentration of long-chain acylcarnitines decreased. Procalcitonin showed better accuracy than conventional indicators in diagnosing severe cholangitis. In addition, acetyl-L-carnitine was independently associated with 28-day mortality. In conclusion, procalcitonin may serve as a specific indicator for predicting the severity of acute cholangitis and the need for biliary drainage, whereas acetyl-L-carnitine may be a potential prognostic factor for patients with acute cholangitis (Table 1).^[44]^

## 3. Discussion

### 3.1. Challenges and future prospects of metabolomics in the study of benign gallbladder diseases

#### 3.1.1. Data processing and analysis

With the advancement of metabolomics technology, researchers can obtain a large amount of complex metabolite data. However, the processing and analysis of these data still pose a challenge. In the future, more efficient and accurate methods for data processing and analysis need to be developed to improve the sensitivity and accuracy of metabolite detection and promote biomarker discovery.^[53]^

Metabolomics has demonstrated potential in detecting links between molecular concentrations and disease status. However, the field is currently in its infancy phase, and numerous studies have been constrained by limited sample sizes and observational designs. Therefore, the current evidence may not possess the necessary strength to establish cause-and-effect relationships or provide detailed explanations of the underlying mechanisms.

Nevertheless, there is a positive outlook that with the advancement of metabolomics research, specifically through collaborative studies involving multiple centers and countries, we will acquire a more profound comprehension of metabolic pathways and their significance in the development of diseases. Cooperative endeavors involving larger groups and varied populations can yield more extensive datasets, thereby increasing statistical strength and applicability. In addition, improvements in analytical techniques and bioinformatics are allowing researchers to examine metabolic profiles more accurately and sensitively, which may reveal new biomarkers and therapeutic targets.

As metabolomics studies advance and improve in methodology, we can anticipate gaining a deeper understanding of how metabolism impacts both health and disease. This may ultimately lead to the development of personalized diagnostic and therapeutic strategies, tailored to individuals’ unique metabolic profiles and underlying pathophysiology. Future efforts should focus on strengthening interdisciplinary collaboration and communication to address the challenges of metabolomics in the study of benign gallbladder disease and to promote technological innovation and application in the field.^[5]^

### 3.2. Development of personalized medicine

Metabolomics offers opportunities for personalized medicine in benign gallbladder disease. By analyzing the metabolites of individual patients, researchers can create more precise diagnosis, treatment, and rehabilitation plans. Future work will focus on further developing the applications of metabolomics in personalized medicine to improve patients’ quality of life and survival expectations.^[54]^

Despite the use of state-of-the-art analytical techniques and advanced bioinformatics tools, metabolomics experiments still encounter many difficulties and pitfalls. Challenges faced by researchers in metabolomics analysis include technical limitations, bioinformatics challenges, and integration with other “omics” sciences. One of the biggest challenges in metabolomics research is data analysis, which can be the most time-consuming phase in the metabolomics workflow and requires close collaboration between analysts, clinicians, and chemometric analysis experts. Integration of metabolomics into clinical practice will depend on establishing standardized protocols for analytical performance and data analysis and developing validated biomarker approaches that are fit for this purpose.^[55]^

Metabolomics can help with the early detection and differential diagnosis of biliary diseases. Metabolomic profiling can detect specific biomarkers or metabolic signatures that indicate disease involvement before clinical symptoms appear. Metabolomics, which analyzes the metabolic profile of bodily fluids such as bile or serum, may detect subtle metabolic changes associated with biliary diseases early on, allowing for timely intervention and treatment.

Furthermore, metabolomics can help in the differential diagnosis of biliary diseases by distinguishing between benign and malignant conditions. Different diseases may have distinct metabolic profiles due to their underlying pathophysiology. Researchers and clinicians can identify unique metabolic patterns associated with each biliary disease by comparing their metabolomic profiles to those of other patients. This information can help distinguish between benign conditions like biliary colic, gallstones, or biliary dyskinesia and malignant ones like cholangiocarcinoma or pancreatic cancer.

Overall, metabolomics has the potential to significantly improve early detection and differential diagnosis in the context of biliary diseases, providing valuable insights for better patient care and outcomes.

## 4. Conclusion

Metabolomics research in benign gallbladder disease faces numerous challenges and future directions, including optimizing data processing and analysis, exploring and validating biomarkers, advancing interdisciplinary research, and developing personalized medicine. By addressing these challenges, researchers can provide more options for the prevention, diagnosis, and treatment of benign gallbladder disease, further improving patients’ quality of life and survival expectations. In the future, metabolomics technology will play an increasingly important role in the research and clinical application of benign gallbladder diseases.

## Author contributions

**Conceptualization:** Yanzhang Du.

**Data curation:** Wennie A. Wijaya.

**Formal analysis:** Yanzhang Du.

**Investigation:** Yanzhang Du.

**Methodology:** Yanzhang Du.

**Project administration:** Yanzhang Du.

**Supervision:** Wei Hui Liu.

**Validation:** Wennie A. Wijaya, Wei Hui Liu.

**Visualization:** Wennie A. Wijaya, Wei Hui Liu.

**Writing – original draft:** Yanzhang Du.

**Writing – review & editing:** Yanzhang Du, Wennie A. Wijaya, Wei Hui Liu.
